# Dental Hints Department

**Published:** 1907-11

**Authors:** B. H. Teague

**Affiliations:** Aiken, S. C.


### ...DENTAL HINTS DEPARTMENT...

CONDUCTED BY B. H. TEAGUE, D. D. S., AIKEN, S. C., TO WHOM ALL CONTRIBUTIONS TO THIS DEPARTMENT SHOULD BE SENT

Plaster is usually not poured thin enough. A thick mix causes bubbles.

A gum forming a pyorrheal pocket should have a V shaped piece scissored from it, opposite each tooth.

Some patients have an abhorrence to anything in the shape of a dry napkin or roll in the mouth and prefer the rubber dam.

If, when inserting, a partial set for a patient with begrimed teeth, the glaze should be ground from the artificials, these will likewise become grimed and assume more nearly the color of the naturals.

First, let me state that the proper proportion of mercury to use with a given alloy is the amount that we should use to amalgamate it, and is not the amount to be left in the filling.—Dr. M. L. Ward, Review.

Soldering Fluid.—The so-called Miller's soldering fluid consists of a solution of phosphoric acid in eight parts of water to which one part each of lactic acid and glycerine have been added.—Dental Era.

Local Anesthetic.—Ethyl chloride as a freezing agent is a safe and reliable local anesthetic. Another is a mixture of equal parts of cocaine and brucine, one per cent. of each in water. Most of the difficulty with cocaine seems to be due to the use of too strong solutions.—Clinical Medicine.

Obtunding Sensitive Dentin.—Chloride of sodium being used in the animal economy to promote endosmosis, why would it not, in solution with cocain, aid in conducting the latter through the dentin.—N. C. Leonard, Dental Headlight.

LOWER PARTIAL PLATES.—In the construction of a lower partial denture I most heartily endorse the use of a wire connecting the two sides. I find it the most satisfactory method of putting in lower partial plates that I ever employed.—L. E. Roach, Dental Review.

FOR CANKER SORE MOUTH.—I have found the full strength aromatic sulphuric acid almost a specific for this condition. I prescribe internally tincture of ferric chloride, gtt v; potassium chlorate, gr iii; water ½ ounce; every three hours in lemonade.—J. E. Powers, Tri-State Dental Journal.

SURPLUS MERCURY.—Do not fear the use of surplus mercury in making the mix. Bear in mind that it is the quantity that you remove, and not what you put in, that is the item of importance. Use an ample supply so that every particle of alloy will be thoroughly mixed with it.—N. K. Garhart, Dental Summary.

CHLORAL HYDRATE.—Chloral hydrate placed in the socket after tooth extraction will often give happy results in the relief of pain, probably due to the fact that it is decomposed by the alkaline blood into chloroform and potassium formate. —Dental Digest.

FOR THE RELIEF OF NAUSEA.—To relieve nausea in the chair, caused by the rubber dam, etc., phenol-sodique is recommended. Dilute one teaspoonful in about half a glass of water and have the patient take a swallow; repeat in ten or fifteen minutes, if need be.—Brief.

Parke, Davis & Co., Detroit, Mich., have put on the market perhaps the best dental hyperdermic syringe. Its points are par excellence, and their local dental anesthetic—Codrenin, a combination of cocaine, chloretone and adrenalin, is all that may be desired in that line,

TOOTH BLEACHING.—If a brown stain persists apply a dilute solution of oxalic acid and quickly wash out cavity with sodium carbonate and plenty of hot water. Desiccate the dentine with hot air syringe, then saturate the interior dentin with white shellac varnish, harden with hot air and fill with white osteo or line it for insertion of porcelain, gold or amalgam.—G. Fisher, British Dental Journal.

Removing a Congested Pulp.—A congested pulp in an anterior tooth is best removed by using crystals of cocain. Dry the cavity thoroughly and partially fill with powdered cocain. Then puncture the pulp with a sharp instrument, letting the serum saturate the cocaine. Force this back into the pulp tissue, anaesthetizing it, so that it can always be removed without the least pain. If hemorrhage is not too profuse, fill at once.—J. P. Buckley, Dental Review.

Antiseptic Spray for the Mouth.—

℞ Tricresol ........................... m. xxx
Oil of Cassia ........................ m. xx
Aq. des. q. s. ad ................... oz. iv

To be used in spray at a temperature of 105 F. Tricresol has three times the disinfecting value of carbolic acid, while it is three times less poisonous; being neutral in reaction it leaves metallic surfaces bright.—H. C. Ferris, Items of Interest.

Pyorrhea Pockets.—No pocket or pouch alongside a tooth must be left to be filled with food or other foreign matter; it must be cut or scraped out, or burnt, so that the tooth can be kept clean. It is better to have the gum fit closely around the neck of the tooth, even though it only covers one-half of it, than to leave a place to be filled with decayed and decaying matter.—A. W. Harlan, Items of Interest.

Hypodermic Injections in the Gums.—A 10 or 20 per cent solution of chloretone in 75 per cent alcohol is valuable as a topical application previous to the use of the hypodermic needle in the gums. The alcohol cuts the mucus and leaves the membrane absolutely clean with resulting sterilization of the field of operation; the anesthetic action of the chloretone insures the minimum of pain.—T. A. Gormley, Dental Register.

Removing Plaster from the Hands.—In removing plaster from the hands after the application of plaster casts, it would be well to remember the fact that syrup of lime is the strongest solution, and that the application of a little sugar to the hands will greatly assist you. The same rule applies to the removal of casts.—Ga. Jour. of Med. Sur.

Extremely Sensitive Cavities.—Complete anaesthesia of hypersensitive dentin is accomplished by the use of the cocain-adrenalin solution, and in some 150 cases there has been no sign of sloughing or any ill effects. To a 1 per cent solution of cocain hydrochlorid I add an equal quantity of a 1:10,000 solution of adrenalin chlorid in normal saline solution, giving ½ per cent cocain with 1:20,000 adrenalin. With a thoroughly sterilized syringe and fresh solution there can be but little danger of sloughing or necrosis.—Val. McDonald, Dental Cosmos.

Removing Glaze from Carborundum Stones.—To renew carborundrum stones that have become glazed from grinding down teeth containing amalgam fillings, place them in a beaker and cover with a fifty per cent solution of nitric acid, allowing them to remain for two or three hours. Remove and place in a strong solution of sodium bicarbonate for several hours that the acid which has been absorbed by the stones may be neutralized.—W. H. Tweedle, Dental Review.

High-Pressure Anaethesia.—You cannot use any agent that will desensitize the dentin or dentinal fibrillae beyond the odontoblastic membrane or cover of the pulp, and expect that pulp to remain alive. I have never seen a case when this method was used that the pulp was not devitalized eventually, and putrescent in the course of six months. —G. W. Cook, Dentists' Magazine.

Officers Elect of Georgia Dental Society.—Prest., T. C. Gibson; 1st vice-pres., C. P. Davis; 2d vice-pres., W. C. Miller; rec'd sect'y., D. H. McNeil; cor. sec'y., D. L. Hill; treasurer, H. R. Jewitt; journal editor, H. H. Johnson.

To Cut Backing for Facings.—For several years I have done as follows: I take a piece of the gummed portion of an envelope and press it down over the pins of my facing, and with shears trim the size of the facing.

I now have a gummed pattern of the exact size the backing should be cut. I now stick this on my gold plate and trim and punch the holes for pins exactly through where they are already punched in the paper. This saves time and gold.—Miles O. Perkins, Amer D. Jour.

Use of the Blue Light.—The contrivance is a simple 16-candle power blue electric light globe arranged in a funnel shaped tin shield which, at its mouth, is about four inches in diameter; this is extended about four inches and has at its end a round blue glass and convex lens. The round blue glass is used to disseminate the blue rays so that the paient may not know the smplicity of the apparatus, and I attribute no especial virtue to the lens. Dr. Watkins claims that in cases of acute abscess, impacted third molars and their associate lesions he has used the blue light with great effect, relieving the pain and causing the disappearance of the inflammatory condition usually attending this form of disease.—Items of Interest.

Difficult Impressions.—One may occasionally have cases where it is difficult if not impossible, to insert an ordinary impression tray sufficiently large to take the impression of the dental arches owing chiefly to the smallness of the external opening.

One can stretch the lips and angles of the mouth to their fullest extent without hurting the patient, if one takes a double piece of note paper, about an inch and a half wide and three inches long, and places that on the right side of patient's mouth, then, as the tray is inserted sideways on the left side, the paper on the other side is made to act as a retractor, perfectly protecting the side of the mouth from abrasion, and permitting the tray to slide in quite easily. —Harry Rose, British Journal of Dental Science.

Method of Taking Plaster Impressions in Partial Cases.—I take an impression without an impression cup. In doing this the first thing is to have the patient rinse the mouth with milk of magnesia. This lubricates the teeth and makes it possible for the plaster to get a close adaptation to the teeth, without sticking to them. If the plaster is mixed so that it will not drop off in chunks, but run off, you can keep on piling up until you have got sufficient surface, so that you will have as much bulk of plaster as you would in an impression cup. After the plaster has become hard it will not readily move out of place, but may easily be cut with a spatula. I slit it half way through in three or four places, and with a broad spatula placed in the full length of the slit, twist it out and spring it apart. The pieces may easily be placed together again after removal.—William H. Taggart, Review.

Soldering—Now you are ready for soldering; the solder having previously been cut into a long strip, dip in liquid flux and apply to the particular part of the bridge you wish to unite, at the same time cutting down the flame so that it will play directly on the extremity of the solder; unite the cusp to the abutments first and then the cusps to each other, and by so doing you will tend to overcome shrinkage by avoiding an excess of solder in any one area. When this has been done, keep feeding your solder in at the lingual edge of the cusps until the desired contour has been acquired. By manipulating the solder in this manner, you can direct it to the exact spot you wish it to flow; there is no "balling up" and no air bubbles are confined, causing a porous piece when completed, and if the slightest care is exercised it is next to impossible to burn the bridge.—Dr. F. E. Logan, Register.

Fixed Bridges.—The tendency to place fixed bridge-work any and everywhere, irrespective of the type of attachment used, and of the number and stability of supporting teeth, is also entirely too common. This class of work is successful only in proportion as it may be inherently strong and judiciously applied, and because of the dynamic requirements, it will be found that "judicious application" will apply far more often to small pieces of from two to five or six teeth than to those in which a greater number are involved.—Dr. H. J. Goslee, Sun.

THE STEP ANCHORAGE AND ITS INDICATIONS.—We are seldom if ever justified in placing a filling in the approximal surface of a bicuspid or molar where the occluding tooth is in position and the occlusion is normal, without securing it by a step anchorage cut as a right angle to the pulpal wall into the occlusal fissure. If this is done, and the step is cut sufficiently broad and deep making that portion slightly broader at the end farthest from the cavity, there will seldom be any movement of the filling to the cavity, but care must be exercised in preparing the step to make it sufficiently deep and broad to insure sufficient filling material to prevent its stretching or breaking by the force of occlusion in the course of mastication.—J. F. Wallace, Dental Era.

SOLIDIFIED FORMALDEHYDE.—While not a new medicament, has not, to our knowledge been reported within the past year. This is produced by heating the aqueous solution in a sandbath with sufficient heat to form a gas, which instead of evaporating, becomes polymerized as soon as the concentration exceed 40 per cent, increasing in density until solid, which when placed in the pulp chamber coming in contact with the heat and moisture of the tooth liberates formaldehyde gas which has almost entirely lost its caustic properties. Having been sealed in the cavity it finds its way to all portions of the root canal producing thorough sterilization.—Items of Interest.

PRO AND CON DEVITALIZATION.—Considerable trouble has arisen from crowning and bridging teeth containing live pulps. The feasibility of sacrificing the pulps in teeth that are to be subsequently used for the abutment of a bridge is of the utmost importance to both the operator and patient. This subject has occasioned much discussion in the profession, and many practitioners, such as Peeso, Harlan, Kirk, Rhein and others favor the devitalization, while other men of good judgment, such as Black, Guilford and Hunt denounce such procedure as not being conducive to the best and most permanent results.—Dr. F. E. Logan, Dental Register.

Mixing Plaster of Paris.—Stirring plaster induces more rapid chemical action, which causes it to expand to a greater extent than if not stirred at all, and that is the secret of mixing plaster successfully. Sulphate of potassium added to plaster will control such expansion naturally as might occur, and bring it down to the minimum point. I have seen it at times remain perfectly dead, that is, with no movement at all when sulphate of potassium is added. The presence of sulphate of potassium softens the plaster, and the density of the set plaster is impaired, and consequently should not be used for models.—J. H. Prothero, Review.

To Re-bake an Inlay.—If for any reason you desire to rebake a porcelain inlay after you have removed the platinum matrix, make an investment of powdered soap stone and thin shellac varnish, while investment is fresh imbed the inlay cavity side down until flush with margins. Dry slowly, and you may fuse as high as you like with no change to margins or shape. This would probably not do for low fusing porcelain, because the shellac would not all burn out and a discoloration would development. The soap stone powder may be made by scraping a fresh mechanic's crayon.—J. M. Evey, Monmouth, Ill., Tri-State Dental Record.

Injured at Clinic.—Dr. J. L. Whinery, a prominent dentist of Des Moines, Ia., is very dangerously ill from the effects of cocain poisoning sustained by having had a tooth removed in a clinic at the recent National Dental Society Convention, at Minneapolis. A toxic condition resulted from the use of the cocain, and vertigo and cerebral congestion have resulted. Apoplexy is feared as a final complication. Dr. Whinery has had trouble with one of his molars for some time, and thought the national convention of his profession a suitable place to have the tooth removed. Cocain was freely injected to deaden the pain and proved disastrous. While on the way home, and while conversing with a friend on the train, the doctor was seized with unconsciousness, which lasted for several minutes. A second similar attack resulted before he reached home, and they have recurred at frequent intervals since.—Des Moines Leader.

Gold and Amalgam in Combination.—In many instances of extreme decay in molars and bicuspids, gold foil will preserve such teeth when it is placed in apposition to amalgam on the dental surfaces, much better than gold will alone. When I see some of these fillings that were put in twenty-five and thirty years ago, standing up well today, I am convinced that there is a field for the combintion of gold and amalgam in the preservation of teeth. I am not sure yet of the true explanation, but my theory is that by putting the two metals together we have a short circuiting of electrical current which almost always resutls in a beautiful oxidation of the baser metal and clean surface of gold, and as a result of this short circuiting there is an electrical impulse which is inimical to the growth of bacteria. That has been proven so conclusively that we can state it as a fact.—Charles P. Pruyn, Review.

"Whited Sepulchers."—When will dentists learn that the old fad of lining a rubber plate with gold or aluminum is of no possible advantage, but on the other hand is simply a delusion making the denture a "whited sepulcher"? The rubber is still there the same non-conductor, with its heat retaining properties, ruining 80 per cent of upper jaws. A 28-guage gold plate, if covered with rubber all over, would have no advantage over rubber alone.

Instead of bringing to the front, every once in a while, these useless methods, the constant effort should be made to stop the use of rubber for permanent work on the upper jaw and substitute aluminum, which is so cheap none should feel compelled to wear the non-conducting material. It is just as easy to fit a metal plate as rubber, and in flat jaws better results are obtained.—L. P. Haskell, Review.

Composition of Platinoid.—The current issue of Ash's Quarterly Circular gives the composition of platinoid as follows:

| | |
|---|---|
| Copper | 60 parts |
| Nickel | 14 |
| Zinc | 24 |
| Tungsten | 2 |
| | 100 —Hiorns |

We lately had a piece of American platinoid analyzed, and here is the report:

| | |
|---|---|
| Copper | 60.50 |
| Nickel | 15.97 |
| Zinc | 23.00 |
| Lead | .12 |
| Iron | .35 |
| Cadmium | .02 |
| Loss | .04 |
| | 100.00 |

The analysts add: The sample contains no trace of platinum, gold, or silver.—C. A. S. & Co.—Cosmos.

Making Gold Bands, Caps, and Crowns Without Solder.—Recognizing the principle that a thin piece of metal will melt in the flame before a thicker, led me to solder bands for crowns by slipping a very thin piece of gold—say from No. 60 to 120 foil—between the edges of the approximating ends of the band, after the ends have been sprung together; then with the blowpipe heat to almost the melting point, and then direct the flame steadily on the thin piece of gold in the joint until it melts. Of course no flux is used before attempting to melt.

For making a cap, fit the band snugly to a thin plate of gold, leaving an edge all around like the brim of a hat; then flux with the band down and plate on top, heat up as before, and run the flame all around, melting the rim up to very near the band, when it will be found that the two pieces are firmly melted together.

For joining a cap to a band in all-gold crowns, adjust the cap to neatly fit the crown by beveling the inner edge of the cap and the outer edge of the band—thus as it were telescoping one into the other; then cut a piece of very thin plate, having it large enough to extend a little all around the joint after it is adjusted between the band and cap, making a large air-hole in the center of the plate; flux and melt as for the cap.

With a little practice this process will be found easier than soldering, and it is almost needless to say it produces a vastly more sightly and stronger result.—Charles Keyes, Cosmos.

Viroinia State Dental Association.—The Virginia State Dental Association held its twenty-sixth annual convention in the Inside Inn, at the Jamestown Exposition, Sept. 9 to 11, with a large number of delegates, representing principally every city in the state in attendance:

Officers of the ensuing year were elected as follows:

President—Dr. Edward Eggleston, Richmond.

First vice-president—Dr. S. A. Lee, Lynchburg.

Second vice-president—Dr. Edgar J. Applewaite, Newport News.

Third vice-president—Dr. F. W. Stiff, Richmond.

Recording secretary—Dr. George M. Keesee, Richmond.

Corresponding secretary—Dr. W. H. Pearson. Hampton.

Treasurer—Dr. W. H. Ewall, Portsmouth.

Executive committee—Dr. W. H. Mosely, South Boston; Dr. William Pilcher, Petersburg, and Dr. J. W. Manning, Norfolk.

Dr. Pearson was elected corresponding secretary to succeed the late Dr. J. Hall Moore, of Richmond, who had held that office in the association from its organization up to the time of his death a few months ago.

Calcium Chlorid as a Hemostatic in Hemorrhage.—The action of calcium chlorid is rapid if given in sufficiently large doses. I remember being called in the middle of the night to see a medical man suffering from hemorrhage after removal of two lower molars. (He was a victim of chronic leucocythemia.)

Naturally he had tried all the remedies he could think of before sending for me, including adrenalin chlorid and pressure, but bleeding had persisted for twelve hours. I ordered him to take twenty grains of calcium chlorid every hour—now I should order it in one-dram doses. The hemorrhage ceased after the third dose.

Before performing any intranasal operation, I always insist on a patient taking full doses of this drug for three days.—A. Stanley Green, Cosmos.

Application of Method to Sensitive Dentine.—For obtunding sensitive dentine, a small quantity of the cocaine solution is taken into cylinder or barrel of syringe, and tapered nozzle placed in drilled aperture. Test to see if there is a perfect joint between tooth and injecting instrument. When contact without leakage has been secured, continued pressure from one to one and one-half minutes will usually be sufficient to send a minute quantity of the obtunding solution through the tubuli onto the surface of the pulp and cause a surface anesthesia of same, cutting off sensation from all parts of the tooth corresponding to the surface of pulp covered by cocaine solution. If pressure is continued long enough, the entire pulp will be surrounded by the obtunding solution.

We find that the obtunding effect is only obtained when pulp is reached by obtundent, and it is then so pronounced that applications of extreme heat and cold are not felt as well as the excavation of cavity. For grinding teeth with living pulps for crowning, for those who are guilty of such practice, proceed the same as for excavating sensitive dentine.—Dr. C. G. Myers, Summary.

Alphozone in Dental Therapeutics.—This drug only recently put on the market is an organic peroxid, a powerful germicide, non-toxic and non-explosive and indicated in all conditions where a germicide can be brought into contact with the pathogenic micro-organisms present. It is a big improvement over the peroxid of hydrogen, as there is no effervescence, consequently no infiltration of the alveolar process and no liability of increased inflammation. It is especially indicated in the treatment of chronic blind abscess. The solution must be prepared fresh. One tablet dissolved in four ounces of water makes practically a 1-1000 solution which is strong enough. After mechanically evacuating as much of the pus as possible use the solution to thoroughly cleanse canals, wrap a broach in cotton, dip in beechwood creosote using this as a vehicle for the powdered alphozone and insert in canals. This dressing can be hermetically sealed in the cavities, but should be changed every twenty-four hours, as the rapidly forming pus oozing down through apex of root soon neutralizes the dressing. I have cured the most obstinate cases with three such treatments and there has been no recurrence of the trouble.—Edmund J. Kelly, Review.

Between the front teeth to be separated, place a piece of binding wire, then, over this the wedge; and after twisting the ends of the wire together over the incisal edge of one of the teeth, you can drive the wedge as hard as desired without its impinging on the gum.—John Warner Keyes, D. M. D.

A Porcelain Catechism.—The crown of all natural teeth are, when in a normal condition, composed, in a sense, of layers of colors. There is a pink or red center, the pulp. Then the next layer is the dentin, which is of about an ivory color. Then all is covered with enamel, the third layer, in which predominate, I would say, white and gray. It seems reasonable to suppose that we can attain the best results by imitating nature, not only in contour and shade, but also in the physical arrangement of the colors. Inlays made with a core of foundation body which is as near as possible the color of the dentin, covered with enamel bodies which are also as near the shade of the natural enamel as possible, should, and will, produce a good appearance, surely much better than those made by attempting to mix the bodies in the endeavor to obtain a mixture of the exact shade of the tooth. Many inlays can be made to match more closely the natural tooth by placing in the bottom of the matrix a little gum enamel, which will give an indefinite, pinkish cast to the foundation body placed over it; or a brown lining in badly discolored teeth, and sometimes a pure white is indicated, like Brewster's cavity lining.—W. A. Coston, Western Dental Journal.

